# “Throwing a rock at their armored tank”: civilian authority and military tobacco control

**DOI:** 10.1186/1471-2458-14-1292

**Published:** 2014-12-17

**Authors:** Quinn Grundy, Elizabeth A Smith, Ruth E Malone

**Affiliations:** Department of Social and Behavioral Sciences, University of California, San Francisco, 3333 California St, San Francisco, CA 94118 USA

**Keywords:** Tobacco control, Military, Public health, Policy, Civilian

## Abstract

**Background:**

Tobacco use is a major cause of chronic disease, disability and death among military personnel and veterans. However, civilian public health and tobacco control advocates have been relatively silent on the issue. Research on the tobacco industry shows a long history of interference in military tobacco policy through relationships with the United States (US) Congress. The military cannot autonomously implement tobacco control, but is subject to Congressional oversight. Thus, the primary obstacles to effective tobacco control in the military are Congressional political opposition and tobacco industry influence, and by extension, a lack of civilian awareness and support in the policy arena.

**Methods:**

As part of a larger project to explore the topic of civilian support for military tobacco control, we analyzed data from focus groups with public health professionals to better understand their sense of agency and authority in regards to military tobacco control. Researchers conducted 4 focus groups with a total of 36 public health professionals at key conferences for those working in public health and tobacco control. Data were coded and the research team developed an interpretive account that captured patterns and variations in the data.

**Results:**

Public health and tobacco control participants shared a sense of futility regarding civilian efforts to engage in military tobacco control. This stemmed from feeling ignorant of military culture and structure, identifying powerful discourses that opposed tobacco control, particularly in a military context, and the very-real presence of the tobacco industry lobby throughout the policy process.

**Conclusions:**

A strong public health voice on military tobacco control might serve to begin problematizing the tobacco industry’s influence in the military policy arena. As the military moves to institute stronger tobacco control policy, public health and tobacco control professionals should work to engage with and aid its efforts from the outside. Only with such civilian side support can the goal of a tobacco free military be realized.

## Background

In 2009, the Institute of Medicine (IOM) called for phasing in a tobacco-free military [1, 2]. This year, to mark the 50^th^ anniversary of the Surgeon General’s report on smoking, the Under Secretary of Defense for Health Affairs released a memorandum in March 2014 calling for active engagement on the issue of military tobacco use, and prompting a review of tobacco policy in the Department of Defense (DoD) [1, 2]. Citing negative impacts on military readiness, harms to individual performance, and premature death for half of all regular users, the Under Secretary specifically called for organization-wide structural reforms related to the sale of tobacco and tobacco use on military installations. Despite state of the art tobacco cessation programs and the successful implementation of smoke free policies such as those implemented in 2010 in the submarine fleet [3, 4], military personnel continue to have significantly higher rates of tobacco use than civilians [5]. Exceeding the civilian estimate of 20.6%, nearly one quarter of military personnel reported using cigarettes in the past 30 days (24.5%), though this estimate ranged by service from 17.2% in the Air Force to 31.9% in the Marine Corps [6]. Consequently, tobacco use is a major cause of chronic disease, disability and death, accounting for about one sixth of all deaths among the DoD population [2]. The recent conflicts in Iraq and Afghanistan were associated with an increase in tobacco use; smoking prevalence is over 50% higher in military personnel who have been deployed than among those who have not [2].

However, until recently, civilian public health and tobacco control advocates have been relatively silent on the issue. In interviews, public health and tobacco control organization leaders revealed a lack of knowledge about military tobacco use and its impact on the military’s mission, and a belief in the “right” to use tobacco [7]. Civilian public health organization leaders also held mistaken beliefs about military policy development and implementation [7], failing to recognize that military culture is shaped by a strict chain of command [8]. Instead, they extrapolated from the civilian policy process, favoring a democratic, non-hierarchic approach to tobacco control. They prioritized cessation efforts, education, and raising tobacco prices, though none recognized that the latter would require congressional approval [7]. Focus groups with civilian public health and tobacco control professionals revealed that, like their organizations’ leaders, the members regarded tobacco use as integral to military culture, and tobacco control as a low priority for military engaged in combat [9]. Both civilian public health leaders and professionals expressed the opinion that civilian advocates could have little impact on military tobacco control policy [7, 9].

Further, within the military, interviewees and focus group participants (including enlisted personnel and their supervisors, installation tobacco control managers, and policy leaders) perceived tobacco use to be an accepted part of military culture, justified by its purported stress-relieving properties [10–12]. Despite evidence suggesting that military personnel across all 4 services who reported using tobacco also reported significantly higher stress levels [13], service-level policy leaders and installation-level tobacco control managers identified tobacco control as a low priority for their leadership and noted environmental factors that promoted tobacco use including the low cost and easy accessibility of tobacco, tolerance of smoke breaks, uneven enforcement of tobacco control policy, especially during deployment, and the prevalence of smoking areas [11, 12]. Military leaders involved in the recent successful banning of smoking aboard submarines identified key factors contributing to this success including strong empirical support, effective framing of the policy as upholding non-smokers’ rights, and the directive stemming from the highest ranks of the organization [4].

However, the military cannot autonomously implement tobacco control policies; the DoD is subject to Congressional oversight. When the Secretary of the Navy recently proposed ending the sale of tobacco in military stores, members of Congress quickly mobilized to oppose the plan [14–16]. Rep. Richard Hudson (R-NC) and 17 other members of congress (16 Republicans and 1 Democrat, largely from tobacco-producing states) wrote a letter to Secretary Mabus urging him to abandon his proposed Navy tobacco policy review [16]. Rep. Duncan Hunter (R-CA), a member of the House Armed Services Committee, disputed that tobacco use harmed troop readiness, and introduced an amendment to the 2015 National Defense Authorization Act, requiring the continued sale of any product currently in inventory in military exchanges or commissaries [17]; the committee approved the amendment and the legislation passed in the House in a largely bipartisan vote (300-119) on December 4, 2014. Following weeks of talks between the House and the Senate Armed Services Committees, lawmakers expect the legislation to pass the Senate by mid December 2014 [18]. Thus, Congress now requires the military to sell tobacco in exchanges and commissaries.

Researchers have documented a long history of interference on the part of the tobacco industry in military and Veterans’ Administration (VA) tobacco control policy [2, 19, 20]. Since 1985, the military has attempted to lower military smoking rates to be at least on par with civilian rates through multiple tobacco control measures, however, these efforts have been repeatedly weakened or rescinded altogether [20]. The tobacco industry has methodically countered each initiative, sometimes working in concert with civilian union leaders and military insiders to urge Congressional representatives to block or weaken proposed measures [20]. For example, attempts to increase cigarette prices at military stores were thwarted by tobacco industry interference in policymaking through exploitation of complex relationships among the DoD, Congress, commissaries and exchanges, mobilizing alliances with the House Armed Services and Morale Welfare and Recreation Committees, framing price raises as an “erosion of benefits”, and exposing internal DoD conflict over commissary pricing policy [19]. These findings suggest that the primary obstacles to effective tobacco control in the military are political opposition and tobacco industry influence, and by extension, a lack of civilian awareness and support in the policy arena [3].

If military leaders are to explore the possibility of a tobacco-free military, they will require the support and expertise of civilian public health and tobacco control communities. Thus, the research question guiding this project was: What are the perceptions of civilian public health and tobacco control advocates and professionals regarding military tobacco control? As part of a larger project to explore the topic of civilian support for military tobacco control, we conducted focus groups with public health professionals to better understand their sense of agency and authority in regards to military tobacco control.

## Methods

### Design and sampling

Researchers conducted 4 focus groups with a total of 36 public health professionals at key conferences for those working in public health and tobacco control: 2 in November 2010 at the American Public Health Association national meeting in Denver, CO [D1 and D2]; and 2 in August 2012 at the National Conference on Tobacco or Health in Kansas City, MO [KC1 and KC2]. Focus groups are moderated group interviews useful for exploring variability in poorly understood phenomena [21, 22]. A convenience sample of participants was recruited through announcements from the conference organizers and flyers in registration areas. Focus groups were conducted on site in the conference facilities. Inclusion criteria were attendance at the conference, English speaking, and age ≥ 18 years. Participants were paid $40. The researchers obtained ethical approval for the study from the university’s Committee on Human Research (CHR 10-01001).

### Procedures

Two researchers, trained in facilitating focus groups, used a standardized protocol with a low moderator involvement approach, which allowed for the spontaneous emergence of unanticipated information [22]. The co-moderators, prior to commencement of the focus group, obtained informed consent individually. Participants consented to audiotaping; identifying information was deleted in transcripts and participants identified by pseudonym. The co-moderators took field notes and prepared debriefing memos following each group. Participants completed a brief demographic questionnaire (Table 1). Following this, participants were asked to discuss their perceptions of military tobacco use, military tobacco control, and what, if any, role civilian public health advocates and professionals might play in the development of military tobacco control policy.

**Table 1:** **Participant demographics**

Group	D1 N = 11	D2 N = 5	KC1 N = 7	KC2 N = 13	Total N = 36
**Age**					
20-29	5	0	0	3	8
30-39	3	1	3	4	11
40-49	1	2	1	1	5
50-59	1	1	1	2	5
60-69	1	1	0	3	5
70-79			1		1
No data			1		1
**Gender**					
Male	2	2	3	4	11
Female	9	3	4	9	25
**Race**					
American Indian/Alaska Native				1	1
Asian	1			1	2
African-American	6	1	1	4	12
White	4	4	6	7	21
**Ethnicity**					
Hispanic	0	0	0	1	1

### Data analysis

The researchers coded verbatim transcripts into thematic categories. All three authors reviewed the transcripts and together generated a set of preliminary codes. QG and ES then coded all transcripts separately to identify additional codes and through discussion, refined existing codes. QG and ES then prepared a coding manual, including working definitions of the codes. QG and ES each re-coded all transcripts using the coding manual and reached consensus regarding ambiguities in coding through discussion. NVivo software [23] was utilized to manage textual data. By iteratively reviewing data under each code, the research team developed an interpretive account that captured patterns and variations in the data. The interpretive account was refined through writing memos, which were developed with the research team. This study adheres to the RATS guidelines of reporting qualitative studies (http://biomedcentral.com/authors/rats).

## Results

Thirty-six public health and tobacco control professionals participated in the focus groups. Their demographic information is summarized in Table 1.

### “Throwing a rock at their armored tank”

Participants shared a sense that the military was impervious to civilian engagement. Brittany [D1], a county public health professional offered,

“My first thought is, is that even an option? I guess what I know of the military makes me think that if it doesn’t come from within the military, that it will not happen. . . it would just be like throwing a rock at their armored tank”.

Many, “intimidated” by the “daunting” task, characterizing it as “an uphill battle”, anticipated that any civilian-initiated efforts in the area of military tobacco control would be met with great resistance on part of military personnel. Andrew [D2], a tobacco control professional believed “there’d be a lot of pushback from the military to have civilian involvement in their policy”. Ashley [D1] concluded, “I don’t know if it could be a civilian problem. I don’t know if we would be allowed to make it our problem”.

They attributed this resistance to civilians’ lack of knowledge and understanding about military life and norms. Elizabeth [KC1], thwarted in her cessation program efforts on a military base, recounted, “the response I got back is, ‘but you don’t know what it’s like to be in the field’”. Patty [KC2], a civilian employee of the military, reinforced Elizabeth’s sense of rejection, explaining, “The issue I see happening is when civilian people try to come into military world, I’m going to tell you, ‘You have no idea’”. Military culture was characterized as being ‘closed’ to civilian outsiders and participants identified both physical and cultural barriers. Elizabeth [KC1] pointed out, “you can’t just walk onto a base anyway” but that also “the time it takes to learn even the military structure and to be accepted and trusted by them and vice versa of the civilians coming in” would be a “huge effort”.

A few participants, however, felt this was insufficient justification for civilian public health’s lack of engagement. Susan [D1], who worked with active duty personnel, acknowledged that public health was “kind of scared of the military”, and suggested that they should be “getting past that, and, like, learning about that culture, just like we would learn about any other culture”. Using desegregation as a precedent, Beckie [D2] argued

“it’s not valid to just say that because they would be resistant means it should continue because it clearly shouldn’t and it clearly doesn’t, or at least we make progress whenever someone outside the military brings attention to an issue that’s wrong. . . There are too many instances where they’ve had to accept something that was different than what they were doing before and they just got on with it and moved on.”

Acknowledging the existing health disparities, Ashley [D1] agreed: “Just because it’s hard to get to doesn’t mean that we should forget about it. I absolutely think there’s a problem that needs to be addressed, whether we’re the biggest part of it or the military is”.

### Public health in “partnership”

Others, however, continued to assert civilians’ lack of authority. They characterized the military as an institution that “takes care of [its] own” and that typically, “they deal with [problems] within”, which contributed to the perception that involvement of civilians in military policy was not only atypical, but discouraged. Stephanie [KC1] thus explained that efforts toward military tobacco control had to “come from within the military but with the partnership of public health. It can’t be just public health driving this all the way to the top”. Many suggested that the role of public health would be to “partner with [the military]”. Ryan [KC2] suggested a cautiously optimistic approach: “I think we have the power to invite, to build those relationships, those partnerships, to start the discussion. It may not go anywhere”. The primary role of public health within these partnerships was “to be really good educators” about the issue of military tobacco use and to bring expertise from programming with other populations. Erin [KC2] explained, “I always try very hard not to “should” on anybody. So I would, I would not see my role as imposing anything on anyone, whether they’re civilian or active military . . . so I just see myself as a helper, not a policy imposer”. Reinforcing the feeling that military tobacco control was beyond the scope of civilian public health, Stephanie [KC1] observed, “there’s so few that are in public health that . . . are veterans that work on this or have family members that are connected to the military at all or even consider the military a part of the population”. One conceivable way of gaining access to military culture was through military families. Participants believed military families were directly and negatively impacted by military tobacco use (in terms of both the health and economic impacts) and saw these families as a feasible point of access to military culture. Barbara [D1], emphasizing the shared civilian status, suggested that public health “make it, you know, more of like a family thing, when they come home, they’re supporting them not to smoke, you know. Because they’re [not] in the military. We’re not either. You know. We’re kind of working together as a team”.

### We’re the tax payers

An alternate construction of civilian authority, however, was as “tax payers”. Depicting a democratic hierarchy, ending in the “tax payer”, Edward [D1], a former colonel in a Medical Service Corps, pointed out that, “The Joint Chiefs of Staff take their orders from a Commander in Chief who, eventually, when you look around, we’re the taxpayers. And we, the civilians, say [what] the military will do or not do”. Civilians’ authority rested on the assertion that as taxpayers, they funded the military, and thus should have a say in its operations. The construction of the taxpayer resonated among participants across groups, evoking concern over the “long-term healthcare cost[s]” for veterans with tobacco-related disease that they perceived to be ultimately shouldered by taxpayers. One participant posed the rhetorical question, signifying this particular logic, “why should I have to pay higher military fees so that the people working there can engage in self-destructive behavior?” They felt that framing the issue of military tobacco control as being cost-effective could motivate civilian groups to engage. However, although they felt that framing military tobacco control in this way could gain traction, they identified no specific mechanism by which the authority of taxpayers could be asserted.

### Policy-level efforts

A minority perspective that arose was the characterization of the military as an institution embedded in a democratic society and thus, theoretically governed by citizens. Bill [KC2], a retired Air Force member explained,

“And also, who runs the military? You do. Not military. That’s why we have a civilian President, a civilian Secretary of Defense . . . Military members happen to have decided to defend the country based on what you want us to do”.

Certain participants wondered if civilians could be more effective at the policy level. Referencing policy directives like the repealing of “Don’t Ask, Don’t Tell”, participants posited, “In the military, once an order is given, it has to be obeyed” [D1]. Beckie [D2] thought:

“There are civilian people who are chiming in on [Don’t Ask, Don’t Tell] and I think that they’re helping to clarify the issues. And then it’s the military people who either have to get on board or not. And the ones who are getting on board are lending credibility to the idea and momentum to the idea that we have to get rid of this policy. But in the end it’s really Congress who’s going to make a decision. And then that’ll be that, you know? There won’t be two ways about it”.

Others disagreed, feeling that Congress was not a feasible target for civilian public health. Participants referenced Congress’s inability to work in a bipartisan manner, the tobacco lobby, and institutional inertia, which served to reinforce the status quo. Noting that tobacco control was a low priority issue for government compared with the economy, for example, they suggested that it would be “political suicide” and that elected officials “wouldn’t want to touch” tobacco control in a military context. Beckie [D2] explained, “I think you definitely don’t want to be caught telling the military what to do, you know? It’s unpatriotic”. Participants reinforced that military tobacco control was beyond their influence by underscoring policy-level challenges in civilian tobacco control: “we, as civilians, can’t even get our FDA to say that this product, which is costing so much in healthcare, and illness and death, is an illegal product. We can’t move that”. Consequently, many participants conceded that any policy-level efforts should be “incremental,” based on “what they’re already doing, and that’s prevention, education, outreach, treatment”.

### Competing with the tobacco industry

In two of the focus groups, there was extensive discussion about the relationship between government and the tobacco industry and these relationships were perceived to have greatly affected the ability of the public health community to influence government on this matter. There was an overall perception that public health professionals could not compete with the resources of the tobacco industry in lobbying for military tobacco control. Ashley [D1], expressing her sense of futility explained,

“The members [of Congress] who come forward to try to kill [smoke free policies] are the people that have received funding from their campaigns from tobacco industry . . .I don’t know if that would ever work, because they get millions of dollars [in campaign contributions] from them. And we can’t compete with millions of dollars. We don’t have millions of dollars”.

Participants perceived that these relationships permeated Congress, the Department of Defense and the Executive Branch. They also understood that these donations constituted relationships that existed over time and that they would not be jeopardized for issues like military tobacco control. Thomas [D1], echoing Ashley’s characterization of the situation, explained,

“And like she said, the Department of Defense, they have a close relationship with the tobacco companies, so it’s not going to be good for them to go against their relationship, try to end their relationship if that’s who’ve been funding you a lot of times over the years”.

In contrast to the longevity of government-industry relationships and the enthusiasm with which the industry pursued and maintained them, John [D2] noted public health’s relative lack of presence in the lobbying arena on the issue of military tobacco control. He remarked that although civilian tobacco control issues were often advocated, on the issue of military tobacco control he did not know of “any concerted advocacy effort that’s been made, you know? If that effort were made to Congress or the particular committees that have oversight responsibilities, maybe there would be a response. I don’t know”. That said, the belief prevailed that “tobacco companies are always going to be the top funder. And the top funder is always going to be the top policy maker in the policies for tobacco use”, suggesting that policy-level efforts would be overshadowed by the industry lobby.

## Discussion

Public health and tobacco control participants expressed a sense of futility regarding civilian efforts to engage in military tobacco control. This stemmed from feeling ignorant of military culture and structure, identifying powerful discourses that opposed tobacco control, particularly in a military context, and the very-real presence of the tobacco industry lobby throughout the policy process. However, participants also challenged this sense of futility, asserting various constructions of civilian agency in regards to military tobacco control policy. Further, participants expressed the importance of addressing health disparities stemming from military tobacco use and a willingness to approach this issue, despite gaps in knowledge and skills.

Focus group participants seemed to be somewhat better informed about the nature of military policymaking than their leadership [7]. Many drew from personal experience working in or with the military, or had friends and family members who had served. Others drew from current events such as the repealing of Don’t Ask, Don’t Tell, to infer that the most successful approach to military tobacco control might be a top-down directive from Congress. This suggests possible avenues for public health leadership to establish contacts and collaboration. However, to date, military tobacco control has not appeared as a public health or tobacco control advocacy priority.

Compounding participants’ insecurity about their role in military health policy was the sense that these efforts would not be welcomed by military personnel or by wider society. They spoke to several discourses that have been effectively furthered by the tobacco industry [24, 25] around military personnel’s “right” to use a legal product and that impinging on tobacco use would be perceived as “unpatriotic”.

One way to frame civilian involvement in military tobacco control that gained traction within the groups was as “taxpayers”. Participants believed that framing military tobacco control in terms of cost-effectiveness could successfully mobilize civilian advocacy, a strategy that is frequently used in public health. For example, APHA recently released a youtube video to promote the value of public health to society: framed in terms of “return on investment”, the narrative equated “saving lives” with “saving money” [26]. In practice, framing military tobacco control solely in economic terms could be challenging, positioning citizens as unwilling to provide for the needs of service members. A recent speech by former Defense Secretary Chuck Hagel, however, suggests a way to do this, using the cost argument as a bridge to the value of health for military members; Hagel referenced the economic costs of military tobacco use, but continued, “Now, the dollars are one thing, but the health of your people, I don’t know if you put a price tag on that. So I think it does need to be looked at and reviewed” [27].

A minority of participants suggested that policy solutions were needed to address the existing health disparities. They suggested the military potential to lead on social justice issues, as they had on desegregation. That said, a number of participants suspected that the true “armored tank” was not the resistance of military personnel, but the tobacco industry’s lobbying efforts, including campaign contributions [28], front groups [25], and the continued promotion of tobacco directly to military personnel [29]. None of the participants identified any public health advocacy regarding military tobacco control in a policy arena.

### Recommendations

Although civilian public health and tobacco control professionals expressed the importance of addressing military tobacco use and the resulting health disparities, significant knowledge gaps existed and participants lacked specific frameworks for action. Civilian public health organizations could begin prioritizing efforts by releasing policy statements regarding military tobacco control; developing sections dedicated to military health research and advocacy; and publishing military health and policy research. As part of efforts to bridge the gaps between civilian and military tobacco control communities, civilian public health organizations might consider building strategic partnerships with military public health and veterans’ service organizations (VSOs). VSOs play important roles in bringing military tobacco control issues to the public’s attention and serve as an active military voice in public policy debates. Despite historic ties to the tobacco industry [25], VSOs have not always supported tobacco industry positions on policy initiatives, and thus, if approached effectively, might be a strong ally in supporting stronger tobacco control policies in the military. Part of this approach should include understanding the messages targeted at veterans and military personnel about tobacco in order to effectively frame initiatives and messages to counter tobacco promotion.

Noting that the military cannot autonomously enact tobacco control policies, civilian public health organizations should target their efforts at Congress. Problematizing the relationships between lawmakers and the tobacco industry could involve publicizing campaign contributions by the tobacco industry to members of Congress [28], lobbying politicians to eliminate tobacco industry contributions, and efforts to raise the status of military tobacco control on the policy agenda through media advocacy.

However, before undertaking this political work, it will be important to develop more effective messaging around the anachronous and inappropriate “right to smoke” discourse that remains an obstacle to the goal of a tobacco-free military. In fact, (a) there is no recognized “right” to smoke; (b) the military tells individuals what they can and cannot do to all the time, including things that have much less impact on their ability to perform (e.g. length of haircut, presence of tattoos); (c) the idea that the military cannot restrict access to an addictive product that damages military performance, increases military costs, and endangers those around them is increasingly inappropriate given the state of the science on tobacco’s actual effects on the military mission.

### Limitations

Focus group participants were a convenience sample, drawn from the population of conference attendees at two major public health and tobacco control conferences. Thus, their perceptions and knowledge of military tobacco control may not be representative of the views of civilian public health and tobacco control professionals. As this was not a purposive sample and no data were collected about participants’ employment or field specialties, it is unknown whether participants were particularly qualified to comment on military tobacco control. However, all participants identified as working in public health or tobacco control and several reported current and/or former involvement with the military in some capacity.

## Conclusions

Although perhaps more “daunting” than liaising with military personnel, a strong public health voice on military tobacco control might serve to begin problematizing the tobacco industry’s influence in the military policy arena. As the military moves to institute stronger tobacco control policy, public health and tobacco control professionals should work to engage with and aid its efforts from the outside. Only with such civilian side support can the goal of a tobacco free military be realized.
